# A prospective analysis of drug-induced acute cutaneous reactions reported in patients at a tertiary care hospital

**DOI:** 10.4103/0253-7613.64487

**Published:** 2010-04

**Authors:** S.C. Hotchandani, J.D. Bhatt, M.K. Shah

**Affiliations:** Professor and Head, Department of Pharmacology, Gujarat, India; 1 Tutor in Pharmacology, Medical college, Vadodara, Gujarat, India

Sir,

An adverse cutaneous drug reaction (ACDR) is an undesirable change in structure and function of the skin, its appendages, or mucous membranes due to drugs.[1] ACDRs are the most common among the various reactions attributed by the drugs.[2] Drug eruptions vary in their appearance, rapidity of onset, severity, and underlying immunopathological mechanisms. They can range from pruritus or rash to severe and life-threatening Stevens Johnson Syndrome or toxic epidermal necrolysis.

The cutaneous eruptions are visible to the patient and hence their reporting is better and early as compared to the drug reactions involving internal organs and systems. Similarly, the response to the treatment for the cutaneous drug reactions is also better perceived. Many of the commonly used drugs can produce ACDRs. Some severe cutaneous ADRs may result in serious morbidity and even death.[3]

In this study, cases of adverse cutaneous drug reactions (ACDRs) were analyzed from both the outpatient and inpatient departments of dermatology at SSG Hospital, Vadodara, Gujarat, to categorize and analyze the reported ACDRs, to evaluate the management and outcome and to assess the causality of the reported ACDRs, and to educate the patients for prevention of their recurrence. Total 70 cases, which had certain, probable or possible relation according to WHO causality assessment criteria, were included in the study. All ACDRs were assessed and analyzed for number of incidences, drug class, individual drug causing ACDRs, and type of cutaneous reactions. Diagnosis and treatment of ACDRs were done by dermatologists of the hospital. We had excluded cases of cutaneous reactions where drugs taken were not known due to patient's unawareness or unavailability of records/prescription. "ADR alert cards" were given to the patients and they were asked to produce the same while visiting the physician in future.

**Figure 1:**
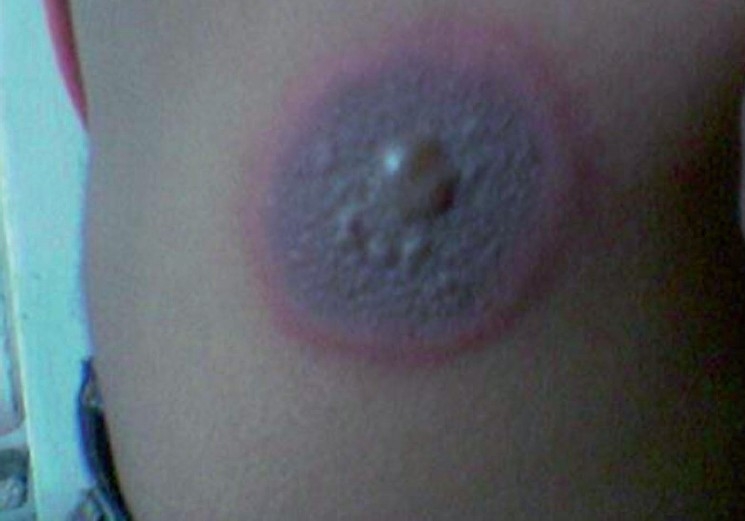
A case of bullous-fixed drug eruption by cotrimoxazole

**Figure 2:**
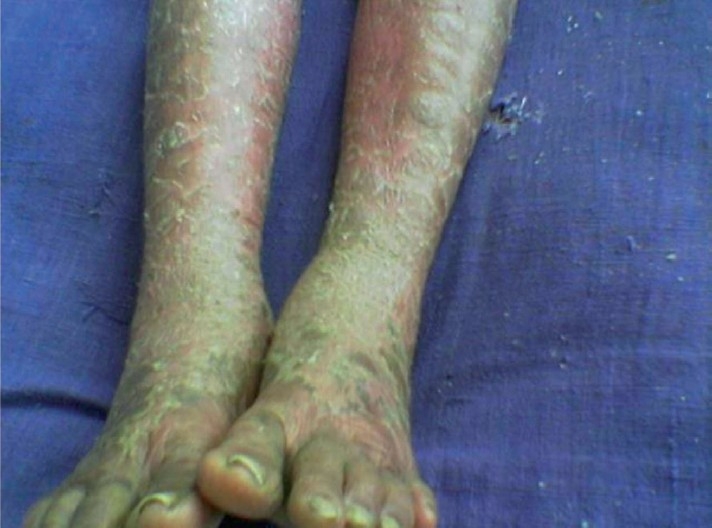
A case of Dapsone syndrome

Most of the patients of ACDRs were in the age group of 11-50 years. Male patients had predominance over female patients. In this study, most cases had reaction time between 1 to 7 days. The most common morphological types of ACDRs were fixed drug eruptions (37.1%) [Figure 1] and maculopapular rash (28.6%). Stevens-Johnson syndrome, dapsone syndrome [Figure 2], and exfoliative dermatitis were the serious ACDRs. Antimicrobials (61.4%), nonsteroidal anti-inflammatory drugs (NSAIDs) (22.9%), and antiepileptic drugs (10%) were the most prominent group of drug responsible for ACDRs.

Pudukadan,[4] Chatterjee *et al*.,[5] and Sharma[6] reported antimicrobials as a major group producing ACDRs followed by antiepileptics and NSAIDs. This indicates regional differences in occurrence of ACDRs probably due to the difference in prescribing patterns of drugs. Maximum number of cases i.e. 15 (21.4%) were due to cotrimoxazole. Among NSAIDs, maximum number (10%) of cases were due to ibuprofen followed by diclofenac (4.3%). Among antiepileptics, phenytoin was implicated in 5 (7.1%) and carbamazepine in 2 (2.9%) cases. ACDRs to some new drugs were also observed like nevirapine-induced maculopapular rash, isotretinoin-induced oral cheilitis, and vitamin B complex-induced maculopapular rash. Out of 70 patients, 68 (97.2%) were cured either due to withdrawal of offending drug or due to appropriate drug therapy to the patients. Among the two cases, one patient developed hyperpigmentation after dechallenge, while the other patient did not return to follow-up after dechallenge. A causal relationship between the drug and the reaction was assessed by using WHO-UMC classification for causality assessment depending upon the lag period between the start of the drug and appearance of the reaction (reaction time), response to dechallenge, and the available data about the drug. According to the WHO-UMC criteria, 9 (12.9%) cases were certain, 59 (84.2%) cases were probable, and 2 (2.9%) of reported ACDRs were possible due to drugs.

Since most of the patients attending this hospital belong to relatively poor socio-economic status, the pattern of drug usage amongst them is mostly restricted to drugs that are supplied free of cost from the hospital. This was an important limitation of this study as the suspected drug information generated from this study may not be truly reflective of the pattern in other health care centers catering to patients of higher socio-economic status.

The observations made in our study emphasize the need for a strict and efficient pharmacovigilance system which could curtail the incidence ACDRs in clinical practice.
